# Bias-Reduced Localization for Drone Swarm Based on Sensor Selection

**DOI:** 10.3390/s25134034

**Published:** 2025-06-28

**Authors:** Bo Wu, Bazhong Shen, Yonggan Zhang, Li Yang, Zhiguo Wang

**Affiliations:** 1 School of Telecommunications Engineering, Xidian University, Xi’an 710126, China; bzshen@xidian.edu.cn (B.S.); zhangyg@sunnyit.com (Y.Z.); 2 Shaanxi Sunny Science and Technology Co., Ltd., Xi’an 710075, China; yanghx@sunnyit.com; 3 School of Computer Science and Technology, Xidian University, Xi’an 710126, China; yangli@xidian.edu.cn

**Keywords:** sensor selection, bias reduction, constraint-weighted least squares (CWLS), frequency difference of arrival (FDOA), time difference of arrival (TDOA)

## Abstract

To address the problem of accurate localization of high-speed drone swarm intrusions, this paper adopts time difference of arrival (TDOA) and frequency difference of arrival (FDOA) measurements, aiming to improve the performance of estimating the motion state of drone swarms. To this end, a two-step strategy is proposed in this study. Firstly, a small number of sensor nodes with random locations are selected in the wireless sensor network, and the constraint-weighted least squares (CWLS) method is used to obtain the rough position and speed information of the drone swarm. Based on this rough information, the objective function of node optimization is constructed and solved using the randomized semidefinite program (SDP) algorithm proposed in this paper to screen out the sensor nodes with optimal localization performance. Secondly, the sensor nodes screened in the first step are used to re-localize the drone swarm, and the CWLS problem is constructed by combining the TDOA and FDOA measurements, and a deviation elimination scheme is proposed to further improve the localization accuracy of the drone swarm. Simulation results show that the randomized SDP algorithm proposed in this paper has the optimal localization effect, and moreover, the bias reduction scheme proposed in this paper can make the localization error of the drone swarm reach the Cramér–Rao Lower Bound (CRLB) with a low signal-to-noise ratio (SNR).

## 1. Introduction

In recent years, with the development of advanced technologies such as artificial intelligence (AI), big data (BD), and the Internet of Things (IoT), drones have been playing an increasingly important role in both national economic development and national defense. In practical drone applications, as the workload and complexity of the tasks they undertake continue to increase, the burden on individual drones in terms of payload, endurance, and decision-making capabilities has been gradually escalating. This has led to the emergence of issues such as weak task execution capabilities, low efficiency, and poor flexibility. The increasing demand for new tasks has driven the development of drone systems towards swarm-based, cost-effective, and miniaturized configurations. Drone swarms have found widespread applications in both civil and military fields [1,2,3,4,5]. However, with the rapid advancement of drone technology, the detection, identification, and tracking of “low, slow, small” drones have become increasingly challenging, making their control and management more complex [6,7]. This is particularly true when drone swarms with unknown origins and fixed geometric configurations intrude at high speeds to conduct reconnaissance and strikes on protected areas. In such cases, ensuring the high-precision localization of drone swarms is crucial to provide necessary information support for subsequent low-altitude safety monitoring and target countermeasures [8]. Classic location techniques include angle of arrival (AOA) [9], time of arrival (TOA) [10], received signal strength (RSS) [11], time difference of arrival (TDOA) [12], and frequency difference of arrival (FDOA) [13]. Among these, AoA and ToA techniques are often used in localization scenarios that require precise angular and temporal estimation due to their high accuracy. For example, [14] proposes a spatial-frequency smoothing technique to efficiently estimate AoA and ToA parameters by “uncorrelating” coherent signals to support a 2D subspace approach. In contrast, TDOA and FDOA techniques do not require a cooperative relationship with the located signal source, giving them a unique advantage in mobile source localization [15].

Localization of high-speed drone swarms typically requires covering a large area with a large number of sensors. However, due to limitations in communication bandwidth and sensor resources, it is generally undesirable to have all sensors measure the signal source simultaneously. This leads to the emergence of the sensor selection problem, which aims to balance the estimation accuracy of the signal source with the number of activated sensors [16,17,18,19]. Selecting a small number of sensors for accurate localization of high-speed moving drone swarms has become a current research hotspot [20,21,22,23]. The sensor selection problem can be viewed as an optimization problem based on different objectives, such as estimation accuracy, energy efficiency, and other key performance indicators [24]. Reference [25] investigated the impact on the multi-objective optimization problem (MOP) when three metrics, Fisher information (FIM), mutual information (MI) and mutual information upper bound (MIUB), were used as the objective function within the sensor selection strategy. Since target localization is essentially a parameter estimation problem, the Cramer–Rao Lower Bound (CRLB) is commonly used to evaluate the performance of drone swarm localization [26].

The sensor selection problem for high-speed dynamic drone swarms can be solved by enumerating all possible sensor combinations to find the optimal solution. However, due to the exponential increase in computational complexity as the number of sensors grows, this approach is not practically feasible. As a result, various optimization algorithms have been proposed, such as the greedy algorithm. The greedy algorithm focuses on selecting the measurement with the largest incremental objective at each step. For general nonlinear models, reference [27] introduced two submodular costs to design the greedy algorithm. In the first step of the algorithm, the nonlinear model is linearized, which results in a performance degradation compared to the convex relaxation solution. In [28], the sensor selection problem is formulated as an integer programming problem, which is then solved using a convex relaxation method. Reference [29] also used convex optimization to solve the problem, with the difference that the authors proposed a problem with continuous placement of sensors based on sensor selection. In practice, sensor deployment in space is subject to errors caused by various factors. In [30], the sensor selection problem under nonlinear measurement models with sensor location error is investigated. Reference [31] proposes the optimization problem of minimizing CRLB sensor selection under the influence of non-line-of-sight errors based on TDOA measurements and the SDR method is used to solve the sensor selection problem for three error measurement cases. In [32], the impact of correlated noise on sensor selection schemes is focused on. A closed-form expression of the FIM with respect to sensor selection variables is derived, which is applicable to sensor selection problems under arbitrary noise correlation conditions. In addition, Refs. [33,34] demonstrate that hybrid measurements can effectively reduce CRLB and propose a sensor selection strategy based on hybrid TOA and RSS measurements, which is shown to result in a significant improvement in both accuracy and complexity. Under different noise situations, a good sensor selection strategy can provide high-precision localization performance for drone swarm control and confrontation while reducing resource consumption. However, the optimization of sensor nodes improves the positioning performance, but bias in the measurement model transformation and solution process will lead to the degradation of positioning accuracy. Therefore how to eliminate the influence of deviation and improve the positioning accuracy of countermeasure drone swarms is the research focus of this paper.

This paper applies the optimization results of sensor selection to construct the localization equations for drone swarms and solve them. Given the dynamic characteristics of drone swarms and their relative motion with respect to pre-deployed sensors, the localization of moving target sources must account for the measurement errors introduced by relative motion. To improve the accuracy of location and velocity estimation for drone swarms, we employ a joint estimation of TDOA and FDOA, and construct corresponding localization equations for motion parameter estimation. Currently, passive localization algorithms based on TDOA and FDOA can be primarily classified into two categories: iterative algorithms and parsing class algorithms. Common iterative algorithms include methods such as Taylor Series expansion [35] and interior point methods [36]. Iterative algorithms are highly dependent on the initial values, and a poor initial point often leads to convergence to a local optimum or failure to converge at all. In contrast, parsing class algorithms do not have convergence issues and offer higher computational efficiency. In [37], a two-stage weighted least squares (TWLS) method was proposed to obtain a closed-form solution for TDOA and GDOA hybrid localization, which has low computational complexity. Due to the relative motion between the drone swarm and the sensors, in [38], TDOA and FDOA measurements were used to construct the localization equation, and the instantaneous velocity and location of the moving source were estimated using the SDR method. The TWLS closed-form solution and SDR method proposed above can achieve localization accuracy that reaches the CRLB under low measurement noise. However, drone swarms often operate in complex environments where measurement noise is large. Overcoming the effects of high noise is crucial for obtaining high-precision localization information for drone swarms. In [39], the localization of a moving source based on TOA and doppler frequency shift (DFS) measurements was studied. Additionally, without neglecting the second-order noise terms, the SDR technique was used to solve the maximum likelihood estimator based on the measurement model. The results showed that this algorithm can achieve better localization performance. Compared with the maximum likelihood estimation, many localization estimators suffer from larger estimation bias. This significant bias limits the accuracy of localization. To address this issue, several bias reduction methods have been developed by researchers. In [40], two bias reduction methods, BiasSub and BiasRed, were proposed for localization based on TDOA and FDOA. Under the assumption of no sensor location errors, the BiasSub method can almost completely eliminate the bias, while the BiasRed method can reduce the bias to the same level as that of the maximum likelihood estimator. In [41], a new bias reduction SDP (BR-SDP) method was proposed for stationary source localization using TDOA and FDOA measurements in the context of mobile sensors. Compared to the traditional SDR method, this new approach significantly reduces the bias in areas with smaller measurement errors. In [42], a bias reduction constraint-weighted least squares (BR-CWLS) method is proposed to solve the localization problem of a moving target in a multistatic scenario with stationary sensors when the carrier frequency is known. In order to reduce the bias generated during the transformation and solution of the measurement model, a bias reduction scheme is introduced in the original SDP method and the error of the weighted matrix approximation is considered for the first time. However, in practical applications, the carrier frequency is unknown. Reference [43] investigates the problem of non-cooperative source localization when the starting transmission time and the carrier frequency are unknown and proposes an SDR method and a BR-SDR method to reduce the bias of the SDR method due to the approximation of the measurement model. Simulation results show that the method can achieve CRLB accuracy.

Table 1 indicates the main differences between this paper and other references. Abbreviations are detailed in the symbol table given in Table 2. Discrete monotonic optimization (DMO) is a sensor selection algorithm proposed in reference [34].

In summary, in the scenario of rapid localization and counteraction against high-speed dynamic incursions by a drone swarm in a monitored area, the challenge is how to select a smaller number of sensors from a large network of deployed sensors, achieving a balance between the localization accuracy of the drone swarm and the resource consumption of the sensor network. Although sensor selection can effectively improve the localization accuracy of a drone swarm, there still exists bias in the localization results. This paper proposes a BR-CWLS method based on TDOA and FDOA measurements, which aims to eliminate the bias during the motion process of the drone swarm and accurately estimate its motion parameters.

Main contributions:

1. In this paper, by jointly utilizing TDOA and FDOA measurements in a 3D scenario, to address the bias problem due to the transformation of the measurement model and the solving process in highly dynamic drone swarm target localization, BR-CWLS is used to reduce the bias effects of the observation matrix and the weighting matrix, and to provide high-precision localization results for drone swarm countermeasures.

2. Localizing a swarm of high-speed moving drones usually requires covering a wide area and a large number of sensor nodes. Aiming at the problem of the impact of different sensor geometric distributions on the performance of realizing target localization of a highly dynamic drone swarm, based on the use of the BR-CWLS algorithm to solve the problem of bias due to the transformation of the measurement model and the solving process, and combining with the stochastic SDP sensor selection algorithm, the optimal sensors are selected to effectively improve the localization accuracy of the drone swarm.

3. This paper provides a comprehensive evaluation and analysis of the relationship between localization accuracy and sensor selection during the high-speed intrusion of an enemy drone swarm. Simulation results show that the proposed randomized SDP algorithm outperforms other algorithms in localization accuracy. When applying the optimization results from the randomized SDP for drone swarm localization, the mean square error (MSE) estimated by BR-CWLS is close to the theoretical accuracy of the CRLB.

The remainder of the paper is organized as follows: Section 2 introduces the drone swarm localization model. In Section 3, we elaborate on the CWLS problem and propose the BR-CWLS algorithm based on it. Section 4 presents the sensor selection problem for the localization of high-speed intrusion by a drone swarm. Section 5 provides simulation verification and result analysis. Finally, Section 6 concludes the paper.

## 2. Problem Description

### 2.1. Drone Swarm Localization System Model

In a three-dimensional scenario, assume there are *M* mobile sensors and *N* drones moving at a constant speed with fixed relative positions in a wireless sensor network.

**Figure 1:**
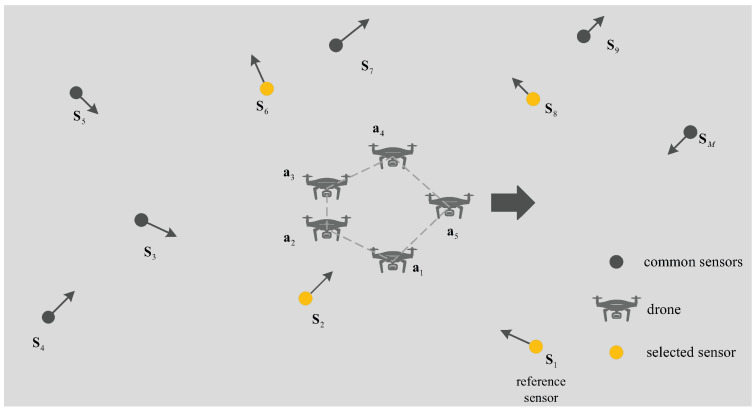
Drone Swarm Localization System Model figure.

As given in Figure 1, $s_{1}$ is set as the reference sensor with a velocity of ${\dot{s}}_{1}$. The other sensors are designated as $s_{2},\ldots ,s_{M}$, with corresponding velocities of ${\dot{s}}_{1},\ldots ,{\dot{s}}_{M}$. The location of the drone swarm is represented by $a_{1},\ldots ,a_{N}$, and its velocity is ${\dot{a}}_{1},\ldots ,{\dot{a}}_{N}$. Without loss of generality, the true TDOA and FDOA between the *m*th sensor and the *n*th drones are

(1) $$t_{m1,n}^{o}=d_{m1,n}^{o}/c,\;m=2,\ldots ,M\;n=1,\ldots ,N,$$

(2) $$f_{m1,n}^{o}=\left(f_{1}{\dot{d}}_{m1,n}^{o}\right)/c,\;m=2,\ldots ,M\;n=1,\ldots ,N.$$

In (2), *c* represents the signal propagation speed, and $f_{1}$ denotes the carrier frequency

(3) $$d_{m1,n}^{o}=∥\begin{array}{c} s_{m}-a_{n} \end{array}∥-∥\begin{array}{c} s_{1}-a_{n} \end{array}∥,$$

(4) $${\dot{d}}_{m1,n}^{o}=\frac{{\left({\dot{s}}_{m}-{\dot{a}}_{n}\right)}^{T}\left(s_{m}-a_{n}\right)}{∥s_{m}-a_{n}∥}-\frac{{\left({\dot{s}}_{1}-{\dot{a}}_{n}\right)}^{T}\left(s_{1}-a_{n}\right)}{∥s_{1}-a_{n}∥}.$$

In practice, TDOA and FDOA measurements are subject to noise as follows:

(5) $$d_{m1,n}=∥\begin{array}{c} s_{m}-a_{n} \end{array}∥-∥\begin{array}{c} s_{1}-a_{n} \end{array}∥+w_{m1,n},$$

(6) $${\dot{d}}_{m1,n}=\frac{{\left({\dot{s}}_{m}-{\dot{a}}_{n}\right)}^{T}\left(s_{m}-a_{n}\right)}{∥s_{m}-a_{n}∥}-\frac{{\left({\dot{s}}_{1}-{\dot{a}}_{n}\right)}^{T}\left(s_{1}-a_{n}\right)}{∥s_{1}-a_{n}∥}+{\dot{w}}_{m1,n}.$$

For simplicity in notation and presentation, the following intermediate variables are defined:

(7) $$r_{i,n}=∥\begin{array}{c} s_{i}-a_{n} \end{array}∥,\;i=1,\ldots ,M,\;n=1,\ldots ,N,$$

(8) $${\dot{r}}_{i,n}=\frac{{\left({\dot{s}}_{i}-{\dot{a}}_{n}\right)}^{T}\left(s_{i}-a_{n}\right)}{∥s_{i}-a_{n}∥},\;i=1,\ldots ,M,\;n=1,\ldots ,N,$$

(9) $$d_{m1,n}^{o}=r_{m,n}-r_{1,n},$$

(10) $${\dot{d}}_{m1,n}^{o}={\dot{r}}_{m,n}-{\dot{r}}_{1,n},$$

(11) $$d_{m1,n}=r_{m,n}-r_{1,n}+w_{m1,n},$$

(12) $${\dot{d}}_{m1,n}={\dot{r}}_{m,n}-{\dot{r}}_{1,n}+{\dot{w}}_{m1,n},$$

where $w_{m1,n}$ and ${\dot{w}}_{m1,n}$ represent the measurement noise of TDOA and FDOA, and $w={\left[\begin{array}{c} w_{21,1},\ldots ,w_{M1,1},\ldots w_{M1,N} \end{array}\right]}^{T}$, $\dot{w}={\left[\begin{array}{c} {\dot{w}}_{21,1},\ldots ,{\dot{w}}_{M1,1},\ldots {\dot{w}}_{M1,N} \end{array}\right]}^{T}$, $w_{n}={\left[\begin{array}{c} w_{21,n},\ldots ,w_{M1,n} \end{array}\right]}^{T}$ and ${\dot{w}}_{n}={\left[{\dot{w}}_{21,n},\ldots ,{\dot{w}}_{M1,n}\right]}^{T}$ are defined as follows. The vector form of all true TDOA and FDOA values is denoted as $d_{n}^{o}={\left[d_{21,n}^{o},\ldots ,d_{M1,n}^{o}\right]}^{T}$ and ${\dot{d}}_{n}^{o}={\left[{\dot{d}}_{21,n}^{o},\ldots ,{\dot{d}}_{M1,n}^{o}\right]}^{T}$, respectively, while the vector form of all TDOA and FDOA measurements is denoted as $d_{n}={\left[d_{21,n},\ldots ,d_{M1,n}\right]}^{T}$ and ${\dot{d}}_{n}={\left[{\dot{d}}_{21,n},\ldots ,{\dot{d}}_{M1,n}\right]}^{T}$. Assume that the measurement noises of TDOA and FDOA both follow a Gaussian distribution with a mean of 0 and are independent of each other. The covariance vectors for the time and frequency domains of the *m*-th sensor for the *n*-th drone are $\left[\sigma_{1,n}^{2},\sigma_{2,n}^{2},\ldots ,\sigma_{M,n}^{2}\right]$ and $\left[{\dot{\sigma }}_{1,n}^{2},{\dot{\sigma }}_{2,n}^{2},\ldots ,{\dot{\sigma }}_{M,n}^{2}\right]$, respectively. The corresponding covariance matrices are as follows:

(13) $$Q_{t,n}=\left(\begin{array}{ccc} \sigma_{2,n}^{2}+\sigma_{1,n}^{2} & \ldots & \sigma_{1,n}^{2}\\ \vdots & \ddots & \vdots \\ \sigma_{1,n}^{2} & \ldots & \sigma_{M,n}^{2}+\sigma_{1,n}^{2} \end{array}\right),$$

(14) $$Q_{f,n}=\left(\begin{array}{ccc} {\dot{\sigma }}_{2,n}^{2}+{\dot{\sigma }}_{1,n}^{2} & \ldots & {\dot{\sigma }}_{1,n}^{2}\\ \vdots & \ddots & \vdots \\ {\dot{\sigma }}_{1,n}^{2} & \ldots & {\dot{\sigma }}_{M,n}^{2}+{\dot{\sigma }}_{1,n}^{2} \end{array}\right).$$

Clearly, the TDOA and FDOA measurement covariance matrix relative to the *n*th drone is denoted as $Q_{n}=bikdiag\left[Q_{t,n},Q_{f,n}\right]$.

### 2.2. Analysis of the Performance Limits of Drone Swarm Localization

The CRLB is the lower bound on the variance of the unbiased estimation of the location parameters in wireless sensor network localization problems, and it serves as an important indicator for evaluating localization performance. Therefore, this paper uses the CRLB as the lower bound for evaluating sensor selection performance, aiming to gain a deeper understanding of its impact on localization accuracy. For simplicity, let $H_{n}={\left[d_{n}^{T},{\dot{d}}_{n}^{T}\right]}^{T}$, n=1,…,N, represent the vectors containing all the measurement values, also corresponding to $\theta_{n}^{o}={\left[a_{n}^{T},{\dot{a}}_{n}^{T}\right]}^{T}$.

To obtain the CRLB for the estimation problem, we first define

(15) $$\rho_{s_{i},a_{n}}=\left(s_{i}-a_{n}\right)/∥s_{i}-a_{n}∥,$$

(16) $$\xi_{s_{i},a_{n}}=\frac{{∥s_{i}-a_{n}∥}^{2}I_{p}-\left(s_{i}-a_{n}\right){\left(s_{i}-a_{n}\right)}^{T}}{{∥s_{i}-a_{n}∥}^{3}}.$$

Without loss of generality, assuming that the noise variance of TDOA and FDOA is independent of the vector $\theta_{n}^{o}$, the FIM for localizing a drone swarm using all the sensors can be expressed as

(17) $${\mathrm{FIM}}_{n}={\left(\frac{\partial H_{n}^{o}}{\partial \theta_{n}^{oT}}\right)}^{T}Q_{m}^{-1}\left(\frac{\partial H_{n}^{o}}{\partial \theta_{n}^{oT}}\right),$$

where

(18) $$\frac{\partial H_{n}^{o}}{\partial \theta_{n}^{oT}}=\left(\begin{array}{cc} \frac{\partial d_{n}^{o}}{\partial a_{n}^{T}} & \;\frac{\partial d_{n}^{o}}{\partial {\dot{a}}_{n}^{T}}\\ \frac{\partial {\dot{d}}_{n}^{o}}{\partial a_{n}^{T}} & \;\frac{\partial {\dot{d}}_{n}^{o}}{\partial {\dot{a}}_{n}^{T}} \end{array}\right),$$

(19) $$\frac{\partial d_{n}^{o}}{\partial a_{n}^{T}}={\left[\rho_{s_{2},a_{n}}-\rho_{s_{1},a_{n}},\ldots ,\rho_{s_{M},a_{n}}-\rho_{s_{1},a_{n}}\right]}^{T},\;\frac{\partial d_{n}^{o}}{\partial {\dot{a}}_{n}^{T}}=0,$$

(20) $$\frac{\partial {\dot{d}}_{n}^{o}}{\partial a_{n}^{T}}={\left[\xi_{s_{2},a_{n}}\left({\dot{s}}_{2}-a_{n}\right)-\xi_{s_{1},a_{n}}\left({\dot{s}}_{1}-a_{n}\right),\ldots ,\xi_{s_{M},a_{n}}\left({\dot{s}}_{M}-a_{n}\right)-\xi_{s_{1},a_{n}}\left({\dot{s}}_{1}-a_{n}\right)\right]}^{T},$$

(21) $$\frac{\partial {\dot{d}}_{n}^{o}}{\partial {\dot{a}}_{n}^{T}}={\left[\rho_{s_{2},a_{n}}-\rho_{s_{1},a_{n}},\rho_{s_{3},a_{n}}-\rho_{s_{1},a_{n}},\ldots ,\rho_{s_{M},a_{n}}-\rho_{s_{1},a_{n}}\right]}^{T}.$$

Taking the derivative of the FIM gives the CRLB for the location and velocity of the *n*th drone:

(22) $${\mathrm{CRLB}}_{n}={\mathrm{FIM}}_{n}^{-1}={\left({\left(\frac{\partial H_{n}^{o}}{\partial \theta_{n}^{oT}}\right)}^{T}Q_{n}^{-1}\left(\frac{\partial H_{n}^{o}}{\partial \theta_{n}^{oT}}\right)\right)}^{-1}.$$

The CRLB for $a_{n}$ and ${\dot{a}}_{n}$ are ${\mathrm{CRLB}}_{n}\left(1:3,\;1:3\right)$ and ${\mathrm{CRLB}}_{n}\left(4:6,\;4:6\right)$, respectively. Then, the CRLB for the drone swarm is

(23) $$\mathrm{CRLB}=bikdiag\{{\mathrm{CRLB}}_{1},{\mathrm{CRLB}}_{2},\ldots ,{\mathrm{CRLB}}_{N}\}.$$

In the next section, we will use the CRLB as the performance metric for the sensor selection algorithm. The objective is to minimize the CRLB in order to select the optimal subset of sensors for localization, thereby improving the localization accuracy of the drone swarm.

## 3. Sensor Selection Algorithm

This paper aims to select a subset of sensors from all available sensors, where each sensor has the potential to be chosen as either a reference sensor or a regular sensor. First, by using the measurement data obtained from *K* sensors and combining it with the CWLS described in Section 4.1, a rough estimate of the drone swarm’s location is calculated. Then, this rough location estimate is used in place of the true location to further construct an optimization problem with the CRLB as the performance metric for the objective function. Finally, by minimizing the objective function, a randomized SDP algorithm is proposed to select the set of sensors that provides the optimal localization performance. This lays the foundation for the subsequent precise determination of the drone’s location.

### 3.1. Sensor Selection Model for Drone Swarm

This paper employs a joint estimation method of TDOA and FDOA, and defines two Boolean vectors

(24) $$p={\left[\begin{array}{c} p_{1},p_{2},\ldots ,p_{M} \end{array}\right]}^{T},\;p_{i}\in \left\{0,1\right\},$$

(25) $$q={\left[\begin{array}{c} q_{1},q_{2},\ldots ,q_{M} \end{array}\right]}^{T},\;q_{j}\in \left\{\begin{array}{c} 0,1 \end{array}\right\},$$

where the *i*th element of q being 1 indicates that $s_{i}$ is selected as the reference sensor, and the *j*th element of p being 1 indicates that $s_{j}$ is selected as the normal sensor. Define the two sensor selection matrices $\Phi_{p}$ and $\Phi_{q}$ generated by p and q. $\Phi_{p}$ is the node selection matrix for locating nodes, obtained by removing all 0 columns corresponding to matrix Diag(p). $\Phi_{q}$ is the sensor selection matrix of the reference node, each column of which is a Boolean vector q, and the number of columns of the matrix is the same as $\Phi_{p}$.

In the classical localization technology, there is only one signal source, but in the current localization fleet there are a total of *N* drones in uniform linear motion. Due to the joint estimation method of TDOA and FDOA, when a single drone in the swarm is located, this requires expanding the two node selection matrices $\Phi_{p}$ and $\Phi_{q}$ along the diagonal.

(26) $$\Phi_{p,n}=\left[\begin{array}{cc} \Phi_{p} & 0\\ 0 & \Phi_{p} \end{array}\right],$$

(27) $$\Phi_{q,n}=\left[\begin{array}{cc} \Phi_{q} & 0\\ 0 & \Phi_{q} \end{array}\right].$$

The Fisher information matrix ${\mathrm{FIM}}_{n}^{sel}$ for the localization of the *n*th drone by the selected sensors is given by

(28) $${\mathrm{FIM}}_{n}^{sel}={\left(\Gamma_{\tau ,n}\Phi_{p,n}-\Gamma_{\tau ,n}\Phi_{q,n}\right)}^{T}{\left(\Phi_{p,n}^{T}\Gamma_{\rho ,n}\Phi_{p,n}+\Phi_{q,n}^{T}\Gamma_{\rho ,n}\Phi_{q,n}\right)}^{-1}\left(\Gamma_{\tau ,n}\Phi_{p,n}-\Gamma_{\tau ,n}\Phi_{q,n}\right),$$

where

(29) $$\Gamma_{\tau ,n}=\frac{1}{c}{\left[\begin{array}{cc} \frac{\partial r_{m,n}^{o}}{\partial a_{n}^{T}} & \;\frac{\partial r_{m,n}^{o}}{\partial {\dot{a}}_{n}^{T}}\\ \frac{\partial {\dot{r}}_{m,n}^{o}}{\partial a_{n}^{T}} & \;\frac{\partial {\dot{r}}_{m,n}^{o}}{\partial a_{n}^{T}} \end{array}\right]}^{T},\;n=1,\ldots ,N,$$

(30) $$\Gamma_{\rho ,n}=Diag\left(\sigma_{1,n}^{2},\ldots .\sigma_{M,n}^{2},{\dot{\sigma }}_{1,n}^{2},\ldots .{\dot{\sigma }}_{M,n}^{2}\right),\;n=1,\ldots ,N.$$

The swarm localization CRLB is obtained by inverting ${\mathrm{FIM}}_{n}^{sel}$ and is used as a performance metric for the optimization problem in Section 3.2. With the objective of minimizing the CRLB, the randomized SDP algorithm is proposed to solve this optimization problem.

### 3.2. Randomized SDP Algorithm Based on Drone Swarm

This section introduces the randomized SDP algorithm for drone swarm localization to improve the performance and computing efficiency of swarm localization problems. In the traditional SDP algorithm, the problem is non-convex because both Boolean constraints and inner products of vectors are non-convex operations. In order to solve this problem, the convex relaxation technique is used to transform the former non-convex problem into a semi-positive definite programming problem. To simplify the matrix inversion, the variable $\Gamma_{\rho ,n}$ is decomposed:

(31) $$\Gamma_{\rho ,n}=\alpha I+\Gamma_{0,n},$$

where α is a constant, I is the identity matrix, and $\Gamma_{0,n}$ is a positive definite matrix. In order to ensure the positive quality of matrix decomposition, α can be set to half of the minimum eigenvalue of the positive definite matrix $\Gamma_{0,n}$. The TDOA and FDOA noise covariance matrix of the selected sensor is represented as

(32) $$\begin{array}{cc} \Gamma_{\rho ,n}^{sel} & =\Phi_{p,n}^{T}\Gamma_{\rho ,n}\Phi_{p,n}+\Phi_{q,n}^{T}\Gamma_{\rho ,n}\Phi_{q,n}\\ \; & ={\left(\Phi_{p.n}+\Phi_{q,n}\right)}^{T}\Gamma_{\rho ,n}\left(\Phi_{p,n}+\Phi_{q,n}\right)\\ \; & =\Lambda +{\left(\Phi_{p,n}+\Phi_{q,n}\right)}^{T}\Gamma_{0,n}\left(\Phi_{p,n}+\Phi_{q,n}\right), \end{array}$$

where $\Lambda =\alpha {\left(\Phi_{p,n}+\Phi_{q,n}\right)}^{T}I\left(\Phi_{p,n}+\Phi_{q,n}\right)$. Substituting $\Gamma_{\rho ,n}^{sel}$ into Equation (28) gives

(33) $${\mathrm{FIM}}_{n}^{sel}=\Gamma_{\tau ,n}\left(\Gamma_{0,n}^{-1}-\Gamma_{0,n}^{-1}{\left(\Gamma_{0,n}^{-1}+C\right)}^{-1}\Gamma_{0,n}^{-1}\right)\Gamma_{\tau ,n}^{T},$$

where

(34) $$C=\left(\Phi_{p,n}-\Phi_{q,n}\right)\Lambda^{-1}{\left(\Phi_{p,n}-\Phi_{q,n}\right)}^{T}=\left[\begin{array}{cc} A & 0\\ 0 & A \end{array}\right],$$

(35) $$A=\alpha^{-1}\left(\mathrm{Diag}\left\{\left(p+q\right)\right\}-\frac{1}{K}\left(p+q\right){\left(p+q\right)}^{T}\right),$$

where the trace of the inverse of ${\mathrm{FIM}}_{n}^{sel}$, that is, the trace of CRLB, is taken as the objective function of the optimization problem, and the constraint conditions are constructed. The constraint condition $1^{T}\left(p+q\right)=K$ represents the selection of *K* sensors, and the constraint condition $p^{T}q=0$ represents the orthogonal Boolean vectors p and q. This means that a node cannot be selected as both a reference node and an ordinary node, and the constraint $p,q\in {\left\{0,1\right\}}^{S}$ indicates that the Boolean vector p and the range of q values are 0 or 1, and the optimization problem can be expressed as

(36) $$\begin{array}{cc} \min\; & \mathrm{trace}{\left({\mathrm{FIM}}_{n}^{sel}\right)}^{-1}\\ s.t.\; & 1^{T}\left(p+q\right)=K,\\ \; & p^{T}q=0,\\ \; & p,q\in {\left\{0,1\right\}}^{S}. \end{array}$$

Let $X=\Gamma_{\tau ,n}\Gamma_{0,n}^{-1}\Gamma_{\tau ,n}^{T}$ and $Y=\Gamma_{\tau ,n}\Gamma_{0,n}^{-1}$. Rewrite problem (36) as follows

(37) $$\begin{array}{cc} \min\; & {\left(X-Y{\left(\Gamma_{0,n}^{-1}+C\right)}^{-1}Y^{T}\right)}^{-1}\\ s.t.\; & 1^{T}\left(p+q\right)=K,\\ \; & p^{T}q=0,\\ \; & p,q\in {\left\{0,1\right\}}^{S}. \end{array}$$

In order to facilitate calculation, an auxiliary matrix Z with the same dimension as FIM is introduced here:

(38) $${\left(X-Y{\left(\Gamma_{0,n}^{-1}+C\right)}^{-1}Y^{T}\right)}^{-1}⪯Z,$$

(39) $$X-Y{\left(\Gamma_{0,n}^{-1}+C\right)}^{-1}Y^{T}⪰Z^{-1},$$

where ⪰ represents the matrix as positive semi-definite. The left side of Equation (38) is CRLB, which can be seen as semi-definite due to the property of CRLB. By inverting the left and right sides of Equation (38), Equation (39) is obtained. $X-Y{\left(\Gamma_{0,n}^{-1}+C\right)}^{-1}Y^{T}$ and Z are known as semi-definite by the property of a positive definite matrix. Because there are two matrix inversion operations in Equation (39), the calculation is more complicated, so a matrix V is introduced.

(40) $$V⪯X-Z^{-1},$$

(41) $$V-Y{\left(\Gamma_{0,n}^{-1}+C\right)}^{-1}Y^{T}⪰0.$$

Furthermore, we define a vector r=p+q and a matrix $R_{r}=rr^{T}$, and A can be expressed as

(42) $$A=\alpha^{-1}\left(Diag\left\{r\right\}-\frac{1}{K}R_{r}\right).$$

Using the Schur complement, the three constraints are transformed into the following linear inequalities:

(43) $$\left[\begin{array}{cc} X-V & I\\ I & Z \end{array}\right]⪰0,\left[\begin{array}{cc} V & Y^{T}\\ Y & \Gamma_{0}^{-1}+C \end{array}\right]⪰0,\left[\begin{array}{cc} R_{r} & r\\ r^{T} & 1 \end{array}\right]⪰0,$$

where the matrix X−V is a positive semidefinite matrix, and the matrix C is a positive semidefinite matrix; when the *i*th elements of both p and q are zero, the *i*th diagonal element of C is zero. However, the last two constraints of these optimization problems remain non-convex. The constraint $p,q\in {\left\{0,1\right\}}^{S}$ can be relaxed by the optimal solutions of the convex constraints $p\in {\left[0,1\right]}^{M}$ and $q\in {\left[0,1\right]}^{M}$. The range of values for p and q is defined between 0 and 1, and the solution to the optimization problem is in fractional form. The constraint $p^{T}q=0$ means that the two elements p and q cannot both be equal to 1, so this constraint is discarded. The constraint problem is rewritten as follows:

(44) $$\begin{array}{cc} \min\; & \mathrm{trace}(Z)\\ s.t.\; & 1^{T}\left(p+q\right)=K,\\ \; & 0\leq q_{i}\leq 1,\;i=1,\ldots ,M,\\ \; & 0\leq p_{j}\leq 1,\;j=1,\ldots ,M,\\ \; & (43). \end{array}$$

Due to the relaxation of the constraint condition $p,q\in {\left\{0,1\right\}}^{S}$, the optimal solution vector is a fractional vector. By examining the fractional vector solutions, a Boolean vector can be obtained. Specifically, in the results of the SDP solution, the Boolean vector can be derived, the fractional vectors $\hat{p}$ and $\hat{q}$ are obtained, and then they are classified as Boolean vectors $\tilde{p}$ and $\tilde{q}$. The specific steps are as follows: for the Boolean vector $\tilde{q}$ of the reference node, the largest fractional value in $\hat{q}$ is selected and set to 1, while the remaining values are set to 0. For the vector $\hat{p}$, the top K−1 largest fractional values are selected and set to 1, with the remaining values set to 0; $\tilde{p}$ and $\tilde{q}$ can serve as the outputs of SDP algorithms. To identify the optimal selection vector, this paper incorporates a randomization technique into the traditional SDP method. The central idea is to generate random fractional vectors related to the SDP solution, which are then used to construct a set of random Boolean selection vectors. The process is as follows: Initially, multiple random Boolean selection vectors are generated from these random fractional vectors. First, a matrix Θ with dimension M∗ε is generated, and ε is the number of randomizations that set the algorithm. The magnitude of the value of each column element of the matrix Θ is set to be 0 to 1, and each column element of this matrix obeys a zero-mean Gaussian distribution. We generate new fractional vectors $\bar{p}$ by summing the fractional vectors generated by the SDP algorithm $\hat{p}$ with each column of the matrix $\sqrt{\left(R_{r}-\left(\hat{q}+\hat{q}\right){\left(\hat{p}+\hat{q}\right)}^{T}\right)}\Theta$ column by column. Similarly, the random vectors $\hat{q}$ and $\sqrt{\left(R_{r}-\left(\hat{p}+\hat{q}\right){\left(\hat{q}+\hat{q}\right)}^{T}\right)}\Theta$ are summed to generate the new fractional vectors $\bar{q}$. Next, the newly generated fractional vectors $\bar{p}$ and $\bar{q}$ are converted to Boolean vectors $\overset{˘}{p}$ and $\overset{˘}{q}$, and at the same time, they are substituted into the objective function, and the corresponding objective function values are recorded. Finally, the Boolean selection vector with the smallest objective function value is selected to determine the optimal sensor subset.

Although the sensors selected by the randomized SDP algorithm can effectively improve the localization accuracy of the drone swarm while saving resources, there still exists a significant bias in the actual localization results. Therefore, this paper will focus on investigating how to reduce the bias in drone swarm localization. In the following, the reference sensors selected by the randomized SDP algorithm will be used as the initial vector for sensor selection, while the remaining sensors will be treated as regular sensors for further selection.

## 4. Bias Reduction Technology

In Section 3, a subset of sensor nodes with the best positioning performance are selected from a large number of available sensor nodes. Based on these deployed sensor nodes, the positioning of the drone swarm is then carried out. In this section, we first utilize the TDOA and FDOA measurement models to formulate a CWLS problem for the initial estimation of the drone swarm’s position and velocity. However, biases occur when constructing and solving the CWLS problem. Therefore, in this section, the idea of bias reduction is further introduced to construct a BR-CWLS problem that implements bias reduction.

### 4.1. Traditional CWLS Problem

In Section 3, the selected sensor subsets are denoted as $s_{k}$ and k=1,2,…,K, where *K* represents the number of selected sensors. To convert the measurement model into a suitable form for algorithm development, we move $r_{1,n}$ to the left-hand side of Equation (9) to obtain $d_{k1,n}^{o}+r_{1,n}=r_{k,n}$. Then, we square both sides of the equation and substitute $r_{k.n}$ from Equation (7) to obtain

(45) $$d_{k1,n}^{o2}-s_{k}^{T}s_{k}+s_{1}^{T}s_{1}=-2{\left(s_{k}-s_{1}\right)}^{T}a_{n}-2d_{k1,n}^{o}r_{1,n}.$$

By differentiating Equation (45), the FDOA equation can be obtained as

(46) $$d_{k1,n}^{o}{\dot{d}}_{k1,n}^{o}+d_{k1,n}^{o}{\dot{r}}_{1,n}+{\dot{d}}_{k1,n}^{o}r_{1,n}={\dot{s}}_{k}^{T}s_{k}-{\dot{s}}_{1}^{T}s_{1}-{\left({\dot{s}}_{k}-{\dot{s}}_{1}\right)}^{T}a_{n}-{\left(s_{k}-s_{1}\right)}^{T}{\dot{a}}_{n}.$$

Let $z_{n}^{o}={\left[a_{n}^{T},{\dot{a}}_{n}^{T},r_{1,n},{\dot{r}}_{1,n}\right]}^{T}$ denote the unknown vector of the n-th drone. By combining Equations (45) and (46), the following result is obtained:

(47) $$\varepsilon_{n}=h_{n}-G_{n}z_{n}^{o},$$

where $\varepsilon_{n}={\left[\varepsilon_{r,n}^{T},\varepsilon_{f,n}^{T}\right]}^{T}$ represents the error vector with neglected second-order noise terms, and $h_{n}={\left[h_{r,n}^{T},h_{f,n}^{T}\right]}^{T}$, $G_{n}={\left[G_{r,n}^{T},G_{f,n}^{T}\right]}^{T}$, where $\varepsilon_{r,n}\approx \left[\begin{array}{cc} B_{1} & 0 \end{array}\right]\lambda_{n}$, $\varepsilon_{f,n}\approx \left[\begin{array}{cc} {\dot{B}}_{1} & B_{1} \end{array}\right]\lambda_{n}$, $\varepsilon_{n}\approx B_{n}\lambda_{n}$, and $\lambda_{n}={\left[\begin{array}{c} w_{21,n},\ldots ,w_{K1,n},{\dot{w}}_{21,n},\ldots ,{\dot{w}}_{K1,n} \end{array}\right]}^{T},$

$$\begin{array}{cc} \; & B_{1,n}=2\left[\begin{array}{cccc} r_{2,n} & 0 & \ldots & 0\\ 0 & r_{3,n} & \ldots & 0\\ \vdots & \vdots & \ddots & \vdots \\ 0 & 0 & \ldots & r_{K,n} \end{array}\right],\;{\dot{B}}_{1,n}=2\left[\begin{array}{cccc} {\dot{r}}_{2,n} & 0 & \ldots & 0\\ 0 & {\dot{r}}_{3,n} & \ldots & 0\\ \vdots & \vdots & \ddots & \vdots \\ 0 & 0 & \ldots & {\dot{r}}_{K,n} \end{array}\right],\;B_{n}=\left(\begin{array}{cc} B_{1,n} & 0\\ {\dot{B}}_{1,n} & B_{1,n} \end{array}\right), \end{array}$$

$$\left.\begin{array}{ccc} h_{r,n}= & \left(\begin{array}{c} d_{21,n}^{2}-s_{2}^{T}s_{2}+s_{1}^{T}s_{1}\\ \vdots \\ d_{K1,n}^{2}-s_{K}^{T}s_{K}+s_{1}^{T}s_{1} \end{array}\right),\;h_{f,n}= & \left(\begin{array}{c} d_{21,n}{\dot{d}}_{21,n}-{\dot{s}}_{2}^{T}s_{2}+{\dot{s}}_{1}^{T}s_{1}\\ \vdots \\ d_{K1,n}{\dot{d}}_{K1,n}-{\dot{s}}_{K}^{T}s_{K}+{\dot{s}}_{1}^{T}s_{1} \end{array}\right)\;, \end{array}\right.$$

$$G_{r,n}=-2\left(\begin{array}{cccc} {\left(s_{2}-s_{1}\right)}^{T} & 0_{3\times 1}^{T} & d_{21,n} & 0\\ \vdots & \vdots & \vdots & \vdots \\ {\left(s_{K}-s_{1}\right)}^{T} & 0_{3\times 1}^{T} & d_{K1,n} & 0 \end{array}\right),$$

$$G_{f,n}=-\left(\begin{array}{cccc} {\left({\dot{s}}_{2}-{\dot{s}}_{1}\right)}^{T} & {\left(s_{2}-s_{1}\right)}^{T} & {\dot{d}}_{21,n} & d_{21,n}\\ \vdots & \vdots & \vdots & \vdots \\ {\left({\dot{s}}_{K}-{\dot{s}}_{1}\right)}^{T} & {\left(s_{K}-s_{1}\right)}^{T} & {\dot{d}}_{K1,n} & d_{K1,n} \end{array}\right).$$

To improve the localization accuracy of the WLS estimator, the relationship between the variables in $z_{n}^{o}$ can be used to formulate the following CWLS optimization problem:

(48) $$\begin{array}{cc} \min_{z_{n}}\; & {\left(h_{n}-G_{n}z_{n}\right)}^{T}W_{n}^{o}\left(h_{n}-G_{n}z_{n}\right),\\ s.t.\; & z_{n}\left(7\right)=∥z_{n}\left(1:3\right)-s_{1}∥,\\ \; & z_{n}\left(8\right)=\frac{{\left(z_{n}\left(1:3\right)-s_{1}\right)}^{T}\left(z_{n}\left(4:6\right)-{\dot{s}}_{1}\right)}{z_{n}\left(7\right)}. \end{array}$$

where $z_{n}$ represents the optimization variable for $z_{n}^{o}$, $W_{n}^{o}$ denotes the weighting matrix, and $W_{n}^{o}={\left(B_{n}Q_{n}B_{n}^{T}\right)}^{-1}$. The constraint in problem (48) is derived from the relationships between the elements of the $z_{n}^{o}$ vector. The solution to the CWLS problem yields ${\hat{z}}_{n}$, from which the estimated values of the source’s position and velocity are obtained as

(49) $${\hat{a}}_{n}={\hat{z}}_{n}\left(1:3\right),$$

(50) $${\hat{\dot{a}}}_{n}={\hat{z}}_{n}\left(4:6\right),$$

(51) $${\hat{r}}_{1,n}={\hat{z}}_{n}\left(7\right),$$

(52) $${\hat{\dot{r}}}_{1,n}={\hat{z}}_{n}\left(8\right).$$

Since the matrix $W_{n}^{o}$ contains the unknown parameters $a_{n}$ and ${\dot{a}}_{n}$, this paper first sets the weighting matrix $W_{n}^{o}$ as $Q^{-1}$. The initial estimates of the unknown parameters $a_{n}$ and ${\dot{a}}_{n}$ are obtained by solving problem (48). Then, the initial estimates of $a_{n}$ and ${\dot{a}}_{n}$ are used to replace the true values in $W_{n}^{o}$ in order to obtain a more accurate solution.

### 4.2. BR-CWLS Algorithm

In the above CWLS method, using the error estimates of $a_{n}$ and ${\dot{a}}_{n}$ to replace the true values in $W_{n}^{o}$ introduces additional bias in the CWLS solution. Moreover, the measurement noise is included in $h_{n}$ and $G_{n}$ of the CWLS algorithm in (48). Additionally, second-order noise terms are ignored when formulating the CWLS problem. These factors contribute to bias in solving the CWLS problem. To reduce this bias, a bias reduction CWLS method is employed in this subsection.

Let D=[G,−h], where $W={\left(\hat{B}Q{\hat{B}}^{T}\right)}^{-1}$ is the weighted matrix containing bias, $\hat{B}$ is the estimated value obtained by the CWLS algorithm replacing the true value in B. Then the matrix D can be decomposed as

(53) $$D_{n}=D_{n}^{o}+\Delta D_{n}=\left[\begin{array}{c} G_{n}^{o},-h_{n}^{o} \end{array}\right]+\left[\begin{array}{c} \Delta G_{n},-\Delta h_{n} \end{array}\right].$$

By replacing $d_{k1.n}$ and ${\dot{d}}_{k1,n}$ in $G_{n}$ and $h_{n}$ with $d_{k1,n}^{o}$ and ${\dot{d}}_{k1,n}^{o}$, respectively, we obtain $G_{n}^{o}$ and $h_{n}^{o}$. After removing the second-order noise, we obtain ΔG and Δh, which are represented as follows:

(54) $$\Delta G_{n}=\varphi_{1,n}J_{1,n}^{o}+\varphi_{2,n}J_{2,n}^{o},$$

(55) $$\Delta h=\varphi_{1,n}\omega_{1,n}^{o}+\varphi_{2,n}\omega_{2,n}^{o},$$

where

$$\varphi_{1,n}=Diag\left\{{\left[w_{21,n},\ldots ,w_{K1,n},w_{21,n},\ldots ,w_{K1,n}\right]}^{T}\right\},$$

$$\varphi_{2,n}=Diag\left\{{\left[{\dot{w}}_{21,n},\ldots ,{\dot{w}}_{K1,n},{\dot{w}}_{21,n},\ldots ,{\dot{w}}_{K1,n}\right]}^{T}\right\},$$

$$J_{1,n}^{o}=\left(\begin{array}{ccc} 0_{(K-1)\times 2p} & -21_{(K-1)\times 1} & 0_{(K-1)\times 1}\\ 0_{(K-1)\times 2p} & 0_{(K-1)\times 1} & -1_{(K-1)\times 1} \end{array}\right),$$

$$J_{2,n}^{o}=\left(\begin{array}{ccc} 0_{(K-1)\times 2p} & 0_{(K-1)\times 1} & 0_{(K-1)\times 1}\\ 0_{(K-1)\times 2p} & -1_{(K-1)\times 1} & 0_{(K-1)\times 1} \end{array}\right),$$

$$\omega_{1,n}^{o}={\left[2d_{21,n}^{o},\ldots 2d_{K1,n}^{o},{\dot{d}}_{21,n}^{o},\ldots ,{\dot{d}}_{K1,n}^{o}\right]}^{T},\;\omega_{2,n}^{o}={\left[0_{(K-1)\times 1}^{T},d_{21,n}^{o},\ldots d_{K1,n}^{o}\right]}^{T}.$$

To separate errors from the inaccurate weighted matrix $W_{n}$, define ${\hat{a}}_{n}=a_{n}+\Delta {\hat{a}}_{n},\;{\hat{\dot{a}}}_{n}={\dot{a}}_{n}+\Delta {\hat{\dot{a}}}_{n}$. Applying the Neumann expansion [43], $W_{n}$ can be approximated as

(56) $$\begin{array}{cc} W_{n} & ={\left({\hat{B}}_{n}Q_{n}{\hat{B}}_{n}^{T}\right)}^{-1}\\ \; & ={\left(\left(B_{n}+\Delta B_{n}\right)Q_{n}{\left(B_{n}+\Delta B_{n}\right)}^{T}\right)}^{-1}\\ \; & ≃{\left(B_{n}Q_{n}B_{n}^{T}+\Delta B_{n}Q_{n}B_{n}^{T}+B_{n}Q_{n}\Delta B_{n}^{T}\right)}^{-1}\\ \; & ≃{\left(B_{n}Q_{n}B_{n}^{T}\right)}^{-1}-{\left(B_{n}Q_{n}B_{n}^{T}\right)}^{-1}\left(\Delta B_{n}Q_{n}B_{n}^{T}+B_{n}Q_{n}\Delta B_{n}^{T}\right){\left(B_{n}Q_{n}B_{n}^{T}\right)}^{-1}\\ \; & =W_{n}^{o}-W_{n}^{o}\Delta P_{n}W_{n}^{o}\\ \; & ≜W_{n}^{o}+\Delta W_{n}, \end{array}$$

where $\Delta W_{n}=-W_{n}^{o}\Delta P_{n}W_{n}^{o}$, $\Delta P_{n}=\Delta B_{n}Q_{n}B_{n}^{T}+B_{n}Q_{n}\Delta B_{n}^{T}$,

(57) $$\Delta B_{n}=\left(\begin{array}{cc} \Delta B_{1,n} & 0\\ \Delta {\dot{B}}_{1,n} & \Delta B_{1,n} \end{array}\right),$$

(58) $$\Delta B_{1,n}=2Diag\left(\frac{{\left(s_{2}-a_{n}\right)}^{T}\Delta {\hat{a}}_{n}}{∥s_{2}-a_{n}∥},\ldots ,\frac{{\left(s_{K}-a_{n}\right)}^{T}\Delta {\hat{a}}_{n}}{∥s_{K}-a_{n}∥}\right),$$

(59) $$\begin{array}{c} \Delta B_{2,n}=2diag(\frac{{\left(s_{2}-a_{n}\right)}^{T}\Delta {\hat{a}}_{n}}{∥s_{2}-a_{n}∥}-\frac{{\left(s_{2}-a_{n}\right)}^{T}\left({\dot{s}}_{2}-{\dot{a}}_{n}\right){\left(s_{2}-a_{n}\right)}^{T}\Delta {\hat{a}}_{n}}{{∥s_{2}-a_{n}∥}^{3}}+\frac{{\left(s_{2}-a_{n}\right)}^{T}\Delta {\hat{\dot{a}}}_{n}}{∥s_{2}-a_{n}∥},\\ \ldots ,\frac{{\left(s_{K}-a_{n}\right)}^{T}\Delta {\hat{a}}_{n}}{∥s_{K}-a_{n}∥}-\frac{{\left(s_{K}-a_{n}\right)}^{T}\left(s_{K}-{\dot{a}}_{n}\right){\left(s_{K}-a_{n}\right)}^{T}\Delta {\hat{a}}_{n}}{{∥s_{K}-a_{n}∥}^{3}}+\frac{{\left(s_{K}-a_{n}\right)}^{T}\Delta {\hat{\dot{a}}}_{n}}{∥s_{K}-a_{n}∥}). \end{array}$$

The method to reduce the deviation is to set the expectation of the second-order error in the objective function as a constant [40]. Therefore, an optimization variable μ is introduced with $y_{n}=\mu {\left[z_{n}^{T}\;1\right]}^{T}$. The objective function is then rewritten in a homogeneous form as $y_{n}^{T}D_{n}^{T}W_{n}D_{n}y_{n}$, and Equations (53) and (56) are substituted into this homogeneous form to obtain

(60) $$\begin{array}{cc} y_{n}^{T}D_{n}^{T}W_{n}D_{n}y_{n} & =y_{n}^{T}{\left(D_{n}^{o}+\Delta D_{n}\right)}^{T}\left(W_{n}^{o}+\Delta W_{n}\right)\left(D_{n}^{o}+\Delta D_{n}\right)y_{n}\\ \; & \;≃y_{n}^{T}D_{n}^{oT}W_{n}^{o}D_{n}^{o}y_{n}+y_{n}^{T}\Delta D_{n}^{T}W_{n}^{o}D_{n}^{o}y_{n}+y_{n}^{T}D_{n}^{oT}\Delta W_{n}D_{n}^{o}y_{n}\\ \; & \;+y_{n}^{T}D_{n}^{oT}W_{n}^{o}\Delta D_{n}y_{n}+y_{n}^{T}\Delta D_{n}^{T}\Delta W_{n}D_{n}^{o}y_{n}\\ \; & \;+y_{n}^{T}D_{n}^{oT}\Delta W_{n}\Delta D_{n}y_{n}+y_{n}^{T}\Delta D_{n}^{T}W_{n}^{o}\Delta D_{n}y_{n}, \end{array}$$

where the third-order error term $y_{n}^{T}\Delta D_{n}^{T}\Delta W_{n}\Delta D_{n}y_{n}$ is ignored when the noise is not particularly large. Under the assumption that the value of $y_{n}$ remains constant, the expectation of Equation (60) can be expressed as

(61) $$\begin{array}{cc} E(y_{n}^{T}D_{n}^{T}W_{n}D_{n}y_{n}) & ≃y_{n}^{T}D_{n}^{oT}W_{n}^{o}D_{n}^{o}y_{n}+E\left(y_{n}^{T}\Delta D_{n}^{T}\Delta W_{n}D_{n}^{o}y_{n}\right)\\ \; & +E\left(y_{n}^{T}D_{n}^{oT}\Delta W_{n}\Delta D_{n}y_{n}\right)+E\left(y_{n}^{T}\Delta D_{n}^{T}W_{n}^{o}\Delta D_{n}y_{n}\right)\\ \; & =y_{n}^{T}D_{n}^{oT}W_{n}^{o}D_{n}^{o}y_{n}+y_{n}^{T}\zeta_{n}^{o}y_{n}, \end{array}$$

where

(62) $$\zeta_{n}^{o}=E\left(\Delta D_{n}^{T}W_{n}^{o}\Delta D_{n}\right)+E\left(\Delta D_{n}^{T}\Delta W_{n}D_{n}^{o}\right)+E\left(D_{n}^{oT}\Delta W_{n}\Delta D_{n}\right).$$

In Equation (61), the mean values of the first-order error terms are zero.

Similarly to $W_{n}^{o}$, $\zeta_{n}^{o}$ in this problem also contains unknown parameter values. To effectively address this issue, this chapter approximates these unknown parameter values by solving the CWLS problem, and subsequently obtains the approximate value of $\zeta_{n}^{o}$, denoted as ${\hat{\zeta }}_{n}$. By fixing the term $y_{n}^{T}{\hat{\zeta }}_{n}y_{n}$ as an arbitrary constant e, the bias is reduced, leading to the derivation of the BR-CWLS problem, which can be expressed as

(63) $$\begin{array}{cc} \min_{y_{n}}\; & y_{n}^{T}D_{n}^{T}W_{n}D_{n}y_{n},\\ s.t.\; & y_{n}\left(7\right)=∥y_{n}\left(1:3\right)-\mu s_{1}∥,\\ \; & y_{n}\left(8\right)=\frac{{\left(y_{n}\left(1:3\right)-\mu s_{1}\right)}^{T}\left(y_{n}\left(4:6\right)-\mu {\dot{s}}_{1}\right)}{y_{n}\left(7\right)},\\ \; & y_{n}^{T}{\hat{\zeta }}_{n}y_{n}=e. \end{array}$$

To prove that *e* in (63) is a positive number, substitute $y_{n}=y_{n}^{o}$ into Equation (61) and obtain

(64) $$0<E\left(y_{n}^{oT}D_{n}^{T}W_{n}D_{n}y_{n}^{o}\right)≃y_{n}^{oT}D_{n}^{oT}W_{n}^{o}D_{n}^{o}y_{n}^{o}+y_{n}^{oT}\zeta_{n}^{o}y_{n}^{o}=y_{n}^{oT}\zeta_{n}^{o}y_{n}^{o}.$$

Since $W_{n}$ is positive definite in (64), it follows that $E(y_{n}^{oT}D_{n}^{T}W_{n}D_{n}y_{n}^{o})>0$, and thus Equation (64) holds. The inequality $y_{n}^{oT}\zeta_{n}^{o}y_{n}^{o}>0$ indicates that $y_{n}^{T}\zeta_{n}^{o}y_{n}>0$ holds when $y_{n}$ approaches the true value of $y_{n}^{o}$. Therefore, when the solution $y_{n}^{*}$ is sufficiently close to the true value of $y_{n}^{o}$, e>0.

Algorithm 1 presents the workflow description of the BR-CWLS positioning algorithm incorporating sensor selection.
**Algorithm 1** BR-CWLS localization algorithm based on sensor selection.**Input:** All sensor positions $s_{m}$; select the number of sensors *K*, observation data matrices $\Gamma_{\tau ,n}$ and $\Gamma_{\rho ,n}$, TDOA and FDOA measurement values, and other known parameters.    Compute problem (44) to obtain the fractional vectors $\hat{p}$ and $\hat{q}$;    Define the maximum index in $\hat{q}$ as 1. Set the remaining indexes to 0 to obtain a new Boolean vector $\tilde{q}$;**for** i=1 to K−1 **do**        $\tilde{p}\left(i\right)=\;\left\{\begin{array}{cc} \;1 & \mathrm{if}\;i=\arg\max(\hat{p})\\ 0 & \mathrm{otherwise} \end{array}\right.$**end for**    The Boolean vectors $\tilde{p}$ and $\tilde{q}$ are output as the result of the SDP. And on the basis of the fractional solutions $\hat{p}$ and $\hat{q}$, a randomization process is introduced;    Generate a random matrix Θ of size M×ε; matrix Θ follows the standard normal distribution.**for** j=1,2,…,ε**do** (ε is the number of randomizations)        Obtain the matrix ${\hat{\Omega }}_{p}$ and ${\hat{\Omega }}_{q}$ with *N* columns of fractional elements through matrix $R_{r}$:        ${\hat{\Omega }}_{p}\left(:,j\right)=\hat{p}+\sqrt{\left(R_{r}-\hat{r}{\hat{r}}^{T}\right)}{|}_{\left(:,j\right)}\Theta$;    ${\hat{\Omega }}_{q}\left(:,j\right)=\hat{q}+\sqrt{\left(R_{r}-\hat{r}{\hat{r}}^{T}\right)}{|}_{\left(:,j\right)}\Theta$;**end for**    Select the fractional vectors $\bar{p}$ and $\bar{q}$ from the matrices ${\hat{\Omega }}_{p}$ and ${\hat{\Omega }}_{q}$ that have the best localization performance. Then, according to steps 2 and 3, convert them into Boolean vectors $\overset{˘}{p}$ and $\overset{˘}{q}$.    Select the corresponding sensors for $\overset{˘}{p}$ and $\overset{˘}{q}$ to locate the drones.    Initialize the weight matrix $W_{n}=Q_{n}$, and estimate the initial estimated value using Equation (48);    Calculate matrix $B_{n}$ with the initial values, update the weight matrix using (9), and obtain a more precise value ${\hat{a}}_{n}$ and ${\hat{\dot{a}}}_{n}$ via (48).    Derive the estimated value of the drones using (63).**Output:** ${\overset{˘}{a}}_{n}$, the position of the nth drone; ${\overset{˘}{\dot{a}}}_{n}$, the speed of the nth drone.

### 4.3. Analysis of Algorithm Complexity

Based on the SDP and randomized SDP algorithms, this section introduces the Best Option Filling (BOF) algorithm, the Iterative Swapping Greedy (ISG) algorithm, the Difference of Convex Functions Programming (DCP), and the Closest Sensors algorithm. From the perspective of computational complexity, we analyze the node optimization selection algorithms for the swarm localization model and evaluate the algorithmic complexity of different optimization selection approaches [31].

In sensor selection algorithms, the exhaustive search method exhibits the highest computational complexity, with the total number of possible sensor subsets being C(M,K)=M!/((M−K)!K!). For instance, in a WSN with 50 sensors, selecting 8 sensors for swarm localization would yield C(50,8) = 4,295,029,200 possible combinations. Due to the necessity of traversing all potential subset combinations, the computational load grows combinatorially, rendering this method impractical for real-world applications, especially in real-time localization scenarios. The Received Signal Strength (RSS)-based closest sensor selection algorithm addresses this issue by selecting the K anchor nodes with the strongest signals for localization. Since the localization model in this study assumes a line-of-sight (LOS) propagation environment, the distance between sensors and the swarm can be directly inferred from RSS—according to electromagnetic wave propagation theory, RSS is inversely proportional to the square of the distance. Although this algorithm achieves low computational complexity, its practical application may suffer from localization accuracy degradation due to RSS measurement errors. By implementing this algorithm, the impact of RSS measurement precision on the performance of rigid-body localization models can be quantitatively analyzed, providing a theoretical foundation for subsequent optimizations.

Let us now analyze the computational complexity of the proposed algorithm and the two methods mentioned above. It should be noted that for each algorithm, since solving the CRLB is unavoidable, we only compare the number of iterations of the algorithms as well as the number of multiplications in computing the CRLB. Specifically, for each computation of the CRLB, $O_{k}=\left(\left(2M+3\right)K^{2}+\left(2M^{2}+6M+9\right)K\right)N$ multiplications are required as well as two matrix inversions, one for the inverse of the covariance matrix $\Gamma_{\rho ,n}^{sel}$ and the other for the inverse of FIM. For the greedy algorithm introduced in this paper, we consider the number of iterations of the algorithm, i.e., the number of times the CRLB is computed. For the ISG algorithm, which can be terminated after a finite number of iterations, the number of iterations is less than the number of selected sensors $N_{ISG}<K$; refer to [31]. For the BOF algorithm, it computes the FIM with a total number of multiplication operations $O_{\mu }=\left(\left(2M+3\right)\mu^{2}+\left(2M^{2}+6M+9\right)\mu \right)N$ per iteration, and μ is the number of sensors per iteration. Overall, the overall complexity of BOF and ISG is $𝒪(M^{2})$. Table 3 summarizes the complexity of the algorithms for comparison.

In SDP-based algorithms, the CVX toolbox by default employs interior-point methods to solve convex optimization problems, with a computational complexity of $𝒪(M^{3})$. While the randomized SDP algorithm builds upon the standard SDP results by incorporating a randomization process (resulting in a longer runtime than standard SDP), it maintains the same asymptotic complexity of $𝒪(M^{3})$.

## 5. Simulation Result

**Figure 2:**
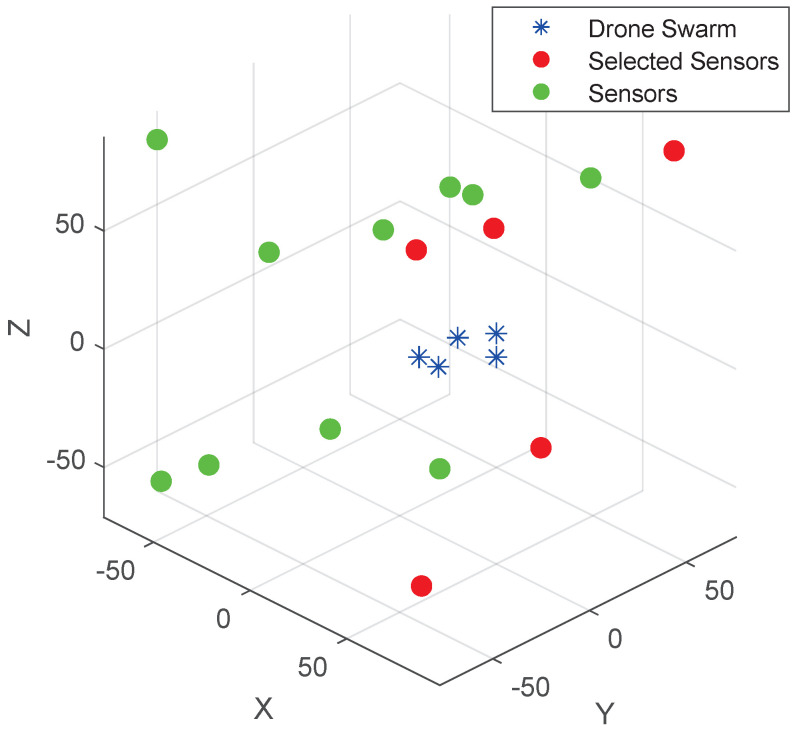
The 3D scenario of the drone swarm (red nodes represent selected sensors, yellow nodes represent unselected sensors, and blue nodes represent the drone swarm).

This section presents the simulation results of using the TDOA and FDOA joint estimation to determine the location of a drone swarm in a three-dimensional wireless sensor network. *M* sensors with random positions are generated within a radius of 1000 m, where each sensor node has a velocity ranging from 0 to 20 m/s. This means that some sensors are stationary while others are mobile. The drone swarm, which needs to be located, is moving at a constant speed of 50 m/s in a straight line in a fixed direction. The distance between any two drones is 3 to 6 m. As shown in Figure 2, it is the distribution of sensor in 3D-space. Table 4 and Table 5 denote the drone swarm coordinates and the main simulation parameters of the drone swarm, respectively.

To validate the noise resistance performance of the proposed sensor node selection algorithm, we deploy 20 sensors in a three-dimensional space; in order to balance the relationship between positioning accuracy and resource efficiency, we selected six sensor nodes with randomized positions to receive the TDOA and FDOA measurement information from the drone swarm. The TDOA and FDOA measurement noises follow a zero-mean Gaussian distribution. And the CRLB is used as the precision metric for evaluating the localization performance of the drone swarm. Regarding the localization performance of the drone swarm, we conducted 1000 simulation environment tests and Monte Carlo simulations, and evaluated the performance of each algorithm.

**Figure 3:**
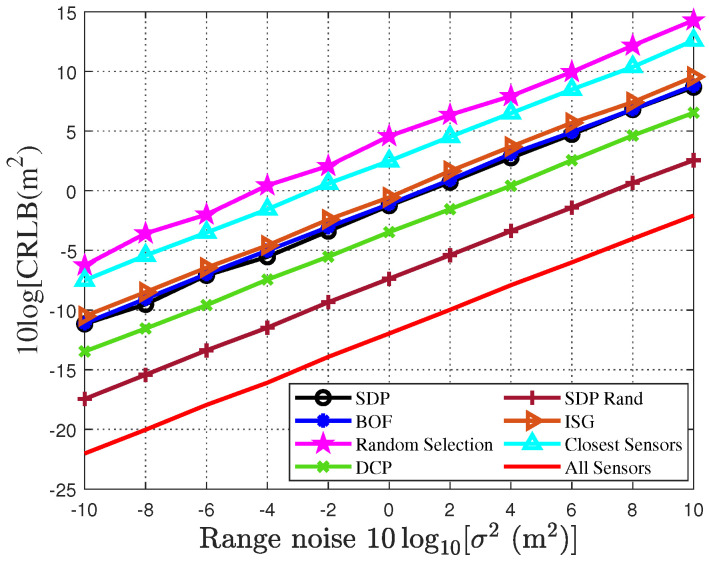
CRLB for sensor selection algorithms for drone swarm localization under different measurement noises.

**Figure 4:**
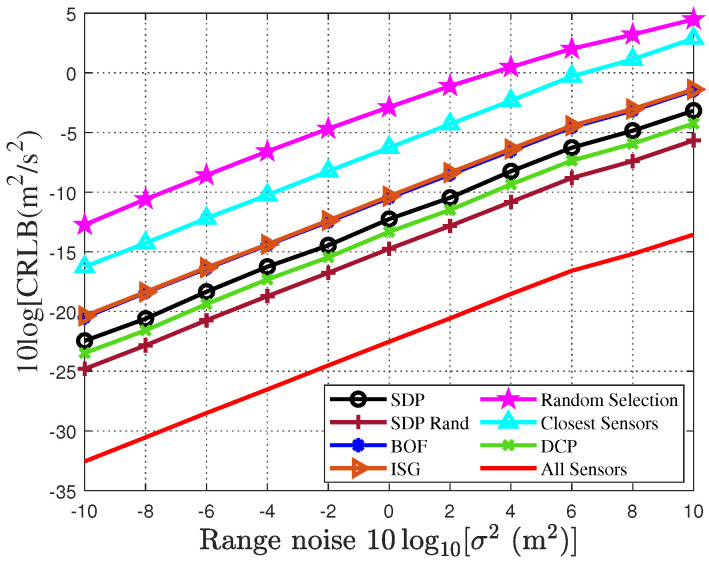
CRLB for sensor selection algorithms to localize drone swarm velocity under different measurement noises.

To validate the impact of the sensor selection algorithms on the localization accuracy of the drone swarm, Figure 3 and Figure 4 present the localization accuracy of various sensor selection algorithms, based on the CRLB as a performance metric, under different noise conditions. Specifically, by setting the variance of the distance measurement noise within the range of −10 dB to 10 dB, we compare the performance of seven different sensor selection algorithms in terms of the localization accuracy of the drone swarm. This analysis helps to reveal the impact of different algorithms on localization accuracy and provides a basis for improving the localization performance of the drone swarm. As shown in Figure 3, with the increase in noise intensity, the localization accuracy of all sensor selection algorithms gradually decreases. Further analysis indicates that under low-signal-to-noise-ratio (SNR) conditions, the localization accuracy of all sensor selection algorithms is relatively low. As the noise level increases, the localization performance of the algorithms shows significant differences: the random selection algorithm and the closest sensors algorithm exhibit the worst accuracy; the traditional SDP algorithm and the two greedy algorithms (BOF and ISG) perform similarly but are consistently outperformed by the randomized SDP algorithm; and the DCP algorithm, though superior to the aforementioned two categories, still underperforms compared to the randomized SDP algorithm proposed in this study. In contrast, the randomized SDP algorithm proposed in this paper demonstrates the best performance in localization accuracy, with its CRLB approaching the accuracy achieved when using 20 sensors for drone swarm localization. Notably, when the noise variance is 10, the CRLB of the randomized SDP algorithm is 1.8 m, while the localization accuracy when using all sensors is 0.62 m. Similarly, in estimating the drones’ intrusion velocity, the randomized SDP algorithm still maintains superior localization performance.

**Figure 5:**
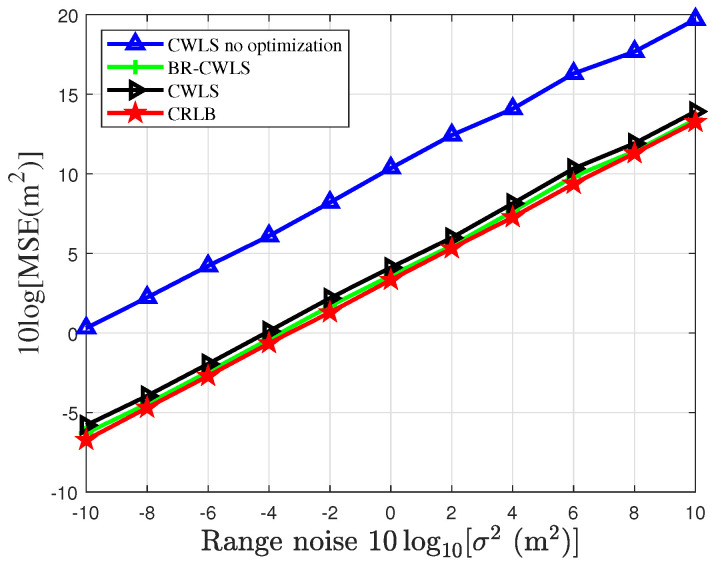
MSE of randomized SDP algorithm optimization results for drone swarm localization under different measurement noises.

**Figure 6:**
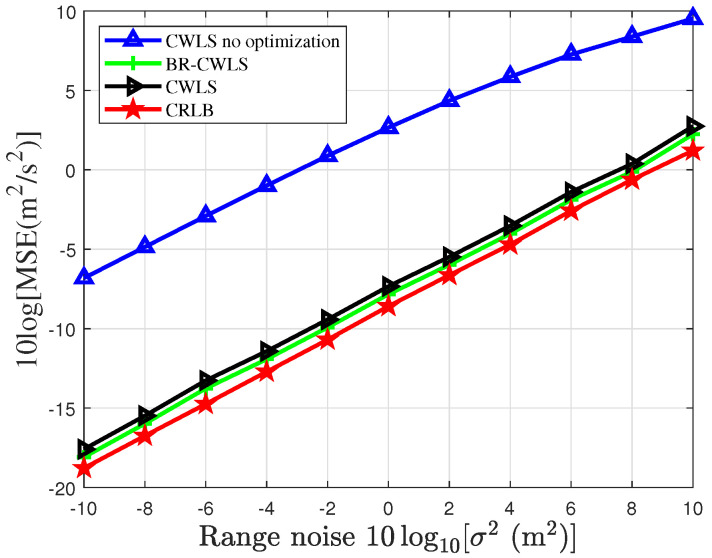
MSE of randomized SDP algorithm optimization results for locating drone swarm velocity under different measurement noises.

To verify the accuracy of the BR-CWLS algorithm in drone swarm localization, Figure 5 and Figure 6 present the results obtained using the randomized SDP algorithm to select 6 sensors from a sensor network with 20 sensors under different noise conditions. In Figure 5, we analyze the impact of node optimization on the localization performance of the drone swarm and compare the localization performance of the BR-CWLS algorithm with that of the traditional CWLS algorithm. This comparison not only helps assess the advantages of the BR-CWLS algorithm but also provides important insights for optimizing drone swarm localization. In Figure 6, we use the optimization results from the randomized SDP algorithm to estimate the velocity of the drone swarm. From the figures, we can observe that the CWLS method without sensor selection algorithm optimization results in the worst localization results, and after applying the sensor selection optimization method, the localization performance significantly improves, with the MSE approaching the CRLB. When the CWLS algorithm employs the bias reduction method, its localization performance is slightly better than that of CWLS without the bias reduction method. However, when the CWLS algorithm without node optimization is used to estimate the drone positions under a noise variance of 0, the average MSE of CWLS is 20.8 m, while the overall average CRLB of the drone swarm is 2.1526 m, and the average MSE of BR-CWLS is 3.6083 m. It can be observed that sensor selection significantly improves the estimation accuracy of both position and velocity for the drones.

**Figure 7:**
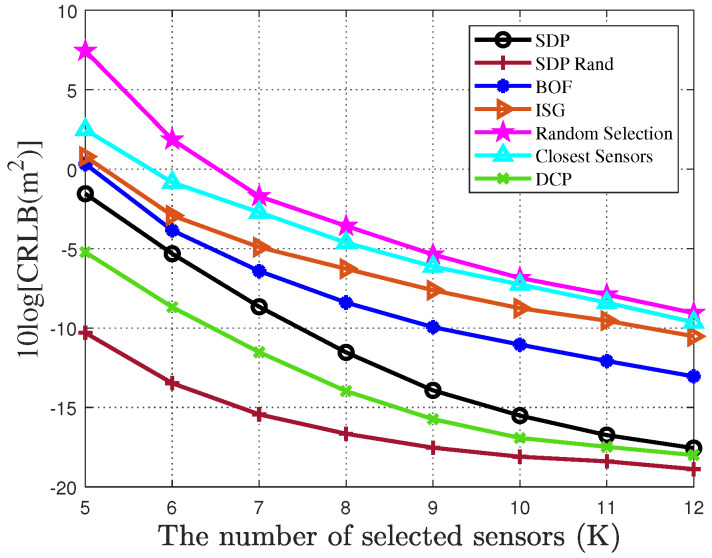
CRLB of sensor selection algorithms for drone swarm localization with different number of sensors.

**Figure 8:**
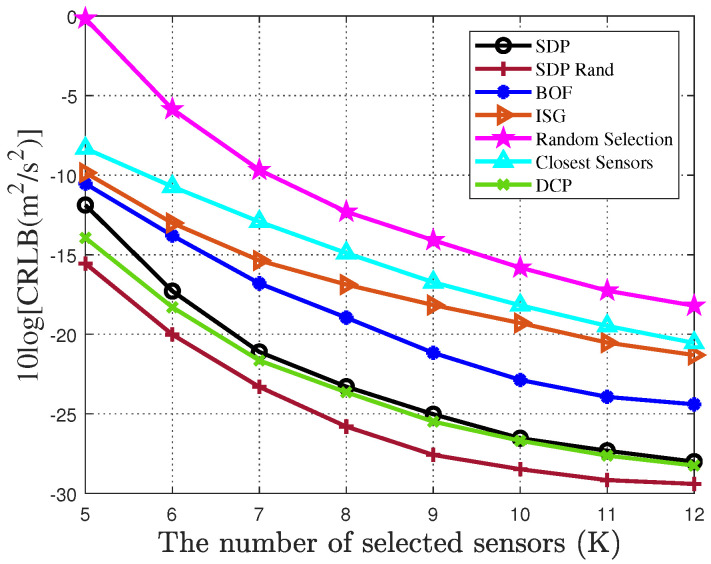
CRLB of sensor selection algorithms for locating drone swarm velocity with different number of sensors.

Figure 7 and Figure 8, respectively, illustrate the performance variations of different sensor selection algorithms for drone swarm localization in a sensor network with 40 sensors under a noise variance of 0 dB. From the figures, it can be observed that as the number of selected sensors increases, the localization performance of all sensor selection algorithms shows a gradual improvement. Among the compared algorithms, both the random selection algorithm and the nearest sensor algorithm demonstrate the poorest initial localization accuracy. Although their performance improves with increasing numbers of selected sensors, they still lag significantly behind other algorithms. A key observation demonstrates that with a limited number of sensors, both SDP and DCP algorithms show significantly lower positioning accuracy compared to the randomized SDP algorithm. However, as sensor quantity increases, their accuracy gradually approaches that of the randomized SDP variant. This phenomenon indicates that while increasing the sensor count enhances localization precision, excessive sensor deployment proportionally raises system energy consumption. The proposed algorithm maintains high-accuracy positioning even with constrained sensor availability, effectively resolving the trade-off between system energy consumption and positioning accuracy. Under 0 dB noise conditions with five selected sensors for drones localization, the randomized SDP algorithm achieves a CRLB of 0.0932 m for position estimation and an average CRLB of 0.0247 m/s for velocity estimation, demonstrating significantly superior performance compared to other algorithms.

**Figure 9:**
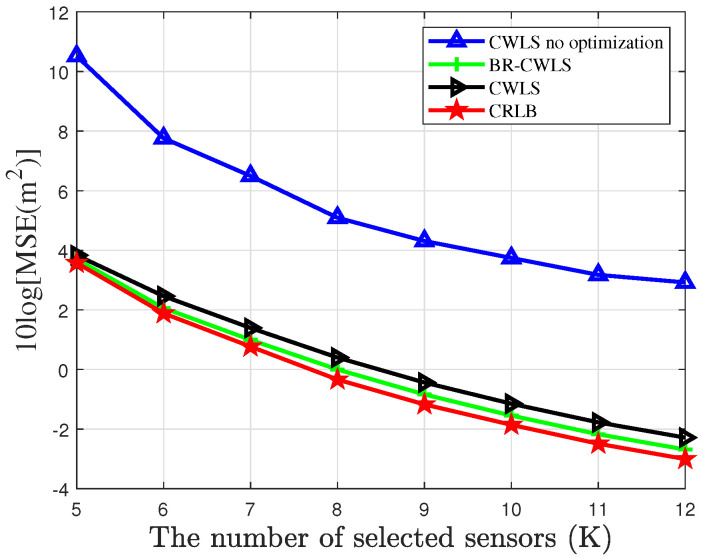
MSE of randomized SDP algorithm optimization results for drone swarm localization with different number of sensors.

**Figure 10:**
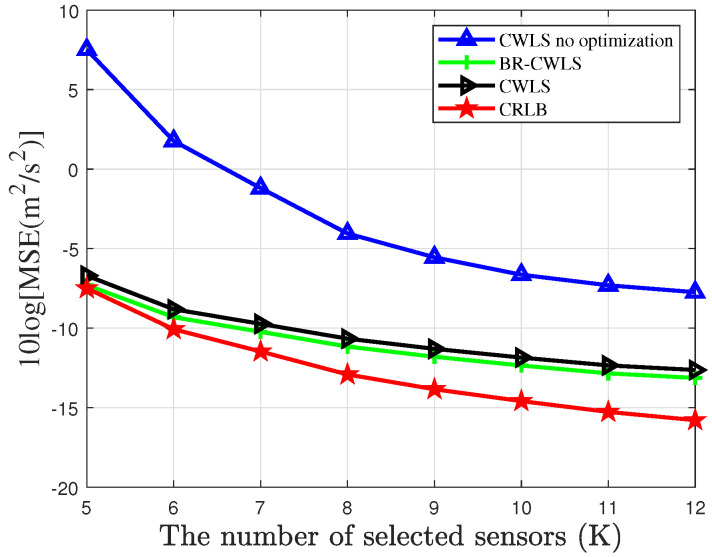
MSE of randomized SDP algorithm optimization results for locating drone swarm velocity with different number of sensors.

Figure 9 and Figure 10 show the results of drone swarm location and velocity estimation using the BR-CWLS algorithm under the condition of noise variance being 0 dB, with different numbers of selected sensors in a network that contains 40 sensors. From the figures, it can be observed that as the number of sensors increases, the localization performance of BR-CWLS for the location gradually improves and remains close to the CRLB. However, during the drone swarm velocity estimation, the results of the BR-CWLS algorithm gradually deviate from the CRLB. It can be observed that for a high-speed moving drone swarm, the estimation of the drone swarm’s velocity still requires further improvement. Finally, it can be seen that when the randomized SDP algorithm is not used for optimization, the localization accuracy of the drone swarm is relatively poor when random sensors from the sensor network are selected.

The proposed randomized SDP and BR-CWLS algorithms are designed to optimize sensor selection and localization accuracy in resource-constrained wireless sensor networks, which are critical for applications such as low-altitude surveillance and target tracking [6,7,8,9]. The simulation parameters include randomly distributed sensors in terms of position and velocity, with TDOA/FDOA measurement parameters following Gaussian distribution, which are consistent with the typical drone swarm localization scenarios described in [31]. While the current study assumes ideal communication conditions, the algorithms’ robustness to varying noise levels (as shown in Figure 5 and Figure 6) suggests their potential applicability in real-world environments with moderate noise variations.

## 6. Conclusions

In this paper, the problem of high-speed drone swarm intrusion into protected areas is discussed, and sensor selection algorithms are investigated to optimize drone swarm positioning. Using TDOA and FDOA techniques, a BR-CWLS algorithm is proposed with the aim of improving the positioning accuracy and efficiency of the sensor network. The localization performance of the randomized SDP algorithm under different noise conditions is verified by simulation. The results show that the algorithm has good localization results. Further analysis shows that the optimization results of the randomized SDP algorithm perform well in drone swarm localization, and the localization accuracy of the BR-CWLS algorithm proposed in this paper is close to that of CRLB. However, the proposed randomized SDP algorithm still has limitations. The paper does not consider the impact on drone swarm localization in complex electromagnetic environments such as non-line-of-sight environments. In future work, we will focus on how to improve drone swarm localization performance using sensor selection algorithms in complex environments.

## Figures and Tables

**Table 1:** The main differences between this paper and other references.

	Sensor	Source	Source Number	Localization Method	Sensor Selection Method
This Paper	Moving	Moving	Multiple	BR-CWLS	Randomized SDP
Reference [31]	Stationary	Stationary	Single	\	SDP
Reference [34]	Stationary	Stationary/Moving	Single	\	DMO
Reference [41]	Stationary	Moving	Single	BR-SDR	\
Reference [42]	Moving	Stationary	Single	BR-CWLS	\

**Table 2:** Description table of main symbols.

Symbol	Description
$a_{n}$, ${\dot{a}}_{n}$	Location and velocity of the *n*th drone swarm
s, $\dot{s}$	Sensor location and velocity in 3D coordinates
${\hat{a}}_{n}$, ${\hat{\dot{a}}}_{n}$	Estimated value of the *n*th drone parameter
$d_{m1,n}$, ${\dot{d}}_{m1,n}$	TDOA and FDOA measurements
$Q_{n}$	Covariance matrix of TDOA and FDOA measurements
p, q	Two boolean vectors
$\Phi_{p}$, $\Phi_{q}$	Sensor node selection matrix
$\Gamma_{\rho ,n}^{sel}$	Measurement noise covariance matrix for selected sensors
M, K	Total number of sensors and number of sensors selected
$\varepsilon_{n}$	Error vector ignoring second-order noise terms
$z_{n}^{o}$	Unknown vector for the *n*th drone
$D_{n}$	Weighting matrices in the presence of error
$W_{n}$	Measurement matrices in the presence of error

**Table 3:** Computational complexity of different algorithms.

Algorithm	Total Number of Operations	Time Complexity
BOF	$\sum_{\mu =4}^{K-1}O_{\mu }$	$O(M^{2})$
ISG	$N_{ISG}K\left(M-K\right)O_{k}$	$𝒪(M^{2})$
DCP	–	$𝒪(M^{3})$
SDP	–	$𝒪(M^{3})$
Randomized SDP	–	$𝒪(M^{3})$
Exhaustive Search	$\frac{M!}{(M-K)!K!}O_{k}$	𝒪(M!)

**Table 4:** Drone swarm coordinates.

	$a_{1}$	$a_{2}$	$a_{3}$	$a_{4}$	$a_{5}$
*x*	3 m	6 m	9 m	3 m	9 m
*y*	0 m	0 m	6 m	6 m	6 m
*z*	0 m	0 m	0 m	0 m	3 m

**Table 5:** Simulation parameters for drone swarm.

Parameters	Size and Scope
Drone swarm velocity	50 m/s
Distance scope per two drones	3–6 m
Number scope of sensors M	20–40
Selection of the number scope of sensors K	5–12
Sensor velocity scope	0–20 m/s
Measurement noise scope	−10–10 dB

## Data Availability

Data is contained within the article.
